# Functional Integration and Segregation in Multiplex Brain Networks for Alzheimer's Disease

**DOI:** 10.3389/fnins.2020.00051

**Published:** 2020-02-18

**Authors:** Lihui Cai, Xile Wei, Jing Liu, Lin Zhu, Jiang Wang, Bin Deng, Haitao Yu, Ruofan Wang

**Affiliations:** ^1^School of Electrical and Information Engineering, Tianjin University, Tianjin, China; ^2^Department of Neurology, Tangshan Gongren Hospital, Tangshan, Hebei, China; ^3^School of Information Technology Engineering, Tianjin University of Technology and Education, Tianjin, China

**Keywords:** integration and segregation, brain network, inter-frequency and temporal dynamics, EEG, Alzheimer's Disease

## Abstract

Growing evidence links impairment of brain functions in Alzheimer's disease (AD) with disruptions of brain functional connectivity. However, whether the AD brain shows similar changes from a dynamic or cross-frequency view remains poorly explored. This paper provides an effective framework to investigate the properties of multiplex brain networks in AD considering inter-frequency and temporal dynamics. Using resting-state EEG signals, two types of multiplex networks were reconstructed separately considering the network interactions between different frequency bands or time points. We further applied multiplex network features to characterize functional integration and segregation of the cross-frequency or time-varying networks. Finally, machine learning methods were employed to evaluate the performance of multiplex-network-based indexes for detection of AD. Results revealed that the brain networks of AD patients are disrupted with reduced segregation particularly in the left occipital area for both cross-frequency and time-varying networks. However, the alteration of integration differs among brain regions and may show an increasing trend in the frontal area of AD brain. By combining the features of integration and segregation in time-varying networks, the best classification performance was achieved with an accuracy of 92.5%. These findings suggest that our multiplex framework can be applied to explore functional integration and segregation of brain networks and characterize the abnormalities of brain function. This may shed new light on the brain network analysis and extend our understanding of brain function in patients with neurological diseases.

## Introduction

As the most common cause of dementia, Alzheimer's disease (AD) is a disabling neurodegenerative disorder characterized by progressive impairment of learning, memory, and other cognitive functions. Earlier studies have suggested that the impairment could arise from focal abnormalities in one or more isolated brain regions such as the entorhinal region or posterior associative cortices (Koenig et al., 2005; Salmon et al., 2005, 2008; He et al., 2009). In contrast, recent neuroimaging studies have shown that the cognitive deficits in AD is related to pathological changes in large-scale functional or structural networks (Dickerson and Sperling, 2005; Greicius, 2008; Sperling et al., 2009; Pievani et al., 2011). Therefore, the investigation of brain networks seems a promising method to study AD pathology. In the past few years, growing attention has been paid to the building of connectivity “neuromarkers” for AD (Toussaint et al., 2012; Franzmeier et al., 2016; Song et al., 2019).

Recent years have also witnessed great advances in neuroimaging modalities such as magneto- and electroencephalography (MEG/EEG), functional magnetic resonance imaging (fMRI), and diffusion tensor imaging (DTI), which provide valuable tools for the identification of networks (Teipel et al., 2009 Ciuciu et al., 2014; Bönstrup et al., 2015; Wu et al., 2017; Dimitriadis et al., 2018). Among the multimodal neuroimaging techniques, EEG may have some major assets from a clinical perspective since it is non-invasive, inexpensive, and easy to use (Fan and Chou, 2018; Jacobs et al., 2018). Compared to most fMRI techniques, the high temporal resolution of EEG also enables the detection of fast neural oscillations that are related to the perception and information exchange between cortical areas (Babiloni et al., 2014; Sigala et al., 2014). Conventionally, EEG connectivity analyses are conducted in different frequency bands separately in AD research, as different EEG rhythms are likely to be involved in different cognitive processes and may be associated with different brain states like waking or sleep (Siegel et al., 2012; Fries, 2015; Wang et al., 2015). Previous studies have reached a consensus with decreased functional connectivity of EEG in alpha band (Yao et al., 2010; Wang et al., 2014; Babiloni et al., 2018). However, EEG network studies may show inconsistent results for other frequency bands (Stam et al., 2006; Chan et al., 2013; Hata et al., 2016). Some researchers reported that AD patients showed higher functional connectivity over centro-parietal regions in the theta band and over occipito-parietal regions in the beta and gamma band (Stam et al., 2006), whereas remarkable reduction of beta connectivity in right frontal region can also be found (Chan et al., 2013). These studies confirmed that the functional networks provide frequency-specific information about the brain function disruptions in AD. In contrast, the diversity in these works also led to a problem whether the frequency-specific functional networks should be analyzed in isolation. Several studies have reported that cross frequency coupling in neural systems support cognitive functions such as memory formation (Tort et al., 2009; Canolty and Knight, 2010; Jirsa and Müller, 2013; Guillon et al., 2017). Therefore, the investigation of the topology by integrating different frequency bands may be a good choice for the network study in AD.

Over the past decade, the graph theory has been widely applied to characterize the network architecture of human brain (Bullmore and Sporns, 2009; Stam, 2014; Storti et al., 2016; Yu et al., 2018). It provides quantitative measurements for each node to depict integrated nature of local brain activity, such as node efficiency and vulnerability. On the other hand, it offers a general language to describe the global properties of brain networks (e.g., modularity). For these reasons, graph theory can provide a promising framework to characterize the pathological processes in AD brain (Tijms et al., 2013; Cai et al., 2018). Recent functional network studies have reported that AD is associated with decreased nodal centrality in higher-order association areas and selective impairment of hippocampus and posterior hub areas (Crossley et al., 2014; Yu et al., 2017). At larger topological scales, the brain networks of AD patients showed increased randomness with a loss of small-world features as the disease progresses (Stam et al., 2007; Sanz-Arigita et al., 2010; Vecchio et al., 2016).

Growing evidence from multimodal neuroimaging techniques have shown abnormalities in functional integration and segregation for AD (Palesi et al., 2016; Kabbara et al., 2018). As two basic properties in human connectome, brain integration, and segregation enable flexible and efficient flow of information within local regions and across the whole brain. Relationships between these properties and the cognitive decline progression were also observed (Kabbara et al., 2018). In addition to the observations in static networks within frequency bands, recent studies have also revealed that the multilayer (cross-frequency) network analysis can provide additional information (i.e., hub) compared to single-layer (frequency-specific) networks (Guillon et al., 2017; Yu et al., 2017). However, most of these studies are focused on the global information processing (integration) but neglected the local information processing (segregation) across frequency bands. Moreover, all these investigations were performed in a static view and the temporal dynamics were not considered in AD. We hypothesized that in AD brain, altered integration and segregation can be found not only across frequency bands but also over time, and such information can be applied to detect AD. Therefore, we construct both cross-frequency and time-dependent networks using multilayer network theory. We provided a common framework to investigate integration and segregation properties in these networks using two multiplex graph metrics. These measures can provide rich information about how information was processed or transferred across frequency bands (time) in global brain or local brain regions. Finally, we tested the diagnostic power of the multiplex network dynamics to discriminate AD patients and healthy controls.

## Materials and Methods

### Participants

Forty subjects are recruited in this study from the neurology department in Tangshan Gongren hospital and divided into two groups: (a) 20 right-handed AD patients (8 males and 12 females, age: 74–78 years) that fulfilled the criteria of probable AD, and (b) 20 age-matched healthy controls (10 males and 10 females, age: 70–76 years). All patients experienced clinical neuroimaging and neurological examination, including structural brain imaging, cerebellar testing, and cranial nerve examination. Exclusion criteria for the patients include use of neuroleptic drugs or antidepressants within 3 weeks before EEG recording and the presence of other neurological or psychiatric conditions and any other severe illness. Similarly, the controls are free from neurological or psychological disorders, alcohol abuse, or any other factor that may affect EEG activity. Mini-Mental State Examination (MMSE) was also implemented to evaluate the cognitive function for both groups. The MMSE scores for the AD patients ranged from 12 to 15, while the scores of healthy controls were distributed between 28 and 30. To avoid interference with the resting-state condition, the cognitive examinations of healthy controls were conducted after the EEG recording. Our study was approved by the local Ethics Committee and the experiments were conducted in accordance with the Declaration of Helsinki. In addition, all the subjects or their legal representatives had been provided with informed consent with the adequate understanding of the purpose and procedure of the study.

### EEG Recordings and Preprocessing

More than 10 min EEG was collected for each subject by a 16-channel Symtop amplifier at a sampling rate of 1024 Hz. Sixteen Ag-AgCl scalp electrodes, Fp1, Fp2, F3, F4, C3, C4, P3, P4, O1, O2, F7, F8, T3, T4, T5, and T6, were set on the scalp according to the international 10–20 system, and the linked earlobe A1 and A2 are used as a reference. During the acquisition, the subject was seated in a semi-dark quiet room and stayed awake with eyes closed. Moreover, they were told in advance to avoid unnecessary body movements or eye blinks. The artifacts were labeled by an experienced researcher and the epochs containing amplitude >80 μV were rejected. In addition, the identification of artifacts was also confirmed by the power spectral density analysis (i.e., the topography of power spectral density). Consequently, four epochs of EEG (50 s long for each) were chosen for each subject. The selected EEG epochs were distributed throughout the whole data except the first 2 min of recordings. Finally, each channel of EEG recordings was decomposed into four sub-bands: delta (1–4 Hz), theta (4–8 Hz), alpha (8–12 Hz), and beta (12–30 Hz) via the finite impulse response (FIR) filter. All procedures were implemented in a MATLAB environment (version 9.1.0.441655, R2016b).

### Multiplex Functional Network Construction

In the construction of multiplex networks, two major factors should be considered: the definition of network edges and layers. In this study, the functional connectivity was estimated by the normalized imaginary part of phase locking values for each layer, which could be denoted by the brain network in a well-known frequency band or a certain time point.

Phase synchrony between two time series within a particular frequency band was assessed by the estimates of the instantaneous phase of the signal ϕ(*t*), which is derived via the Hilbert transform. According to the widely held view, two signals are connected if they have a stable phase difference. Hence, Lachaux et al. defined the phase locking values (PLV) as a time-dependent connectivity measure (Lachaux et al., 1999):

(1) $$\begin{array}{l} PLV_{k,m}=\frac{1}{T}|\overset{T}{\underset{i\;=\;1}{\sum }}e^{-i\left(\varphi_{k}\left(t\right)-\varphi_{m}\left(t\right)\right)}| \end{array}$$

where *T* is the data length. Correspondingly, the imaginary part of PLV, termed imaginary PLV (iPLV), is defined as follows:

(2) iPLVk,m=1T|Im∑i = 1Te-i(φk(t)-φm(t))|

where Im(*x*) denotes the imaginary part of *x*. The iPLV is only sensitive to the non-zero phase lags and is thus less susceptible to the volume conduction effects. However, this measure is not normalized as its upper bound is |sin(φ)| when two signals have a stable phase difference φ. The normalized iPLV (niPLV) can be obtained by the following correction (Bruña et al., 2018):

(3) iPLVk,m=1T|Im∑i=1Te−i(φk(t)−φm(t))|/1−(1T|Re∑i=1Te−i(φk(t)−φm(t))|)2

where Re(*x*) denotes the real part of *x*. When two signals are completely connected with a stable phase difference, niPLV reaches the upper bound 1. Therefore, it is a symmetrical measure and ranges between 0 and 1, with higher values indicating stronger functional interactions.

The frequency-based multiplex (cross-frequency) networks were reconstructed by integrating the four frequency-specific networks, where each layer shared the same set of nodes, but the edges in each layer were defined by the niPLV-weighted functional connections within each frequency band (i.e., delta band). Only the interactions between the same set of nodes across layers are allowed, so the cross-frequency couplings between different brain regions were not considered. In addition to the four-layer networks including all frequency bands, we also considered the interrelationship of two frequency components to reconstruct two-layer networks (i.e., δ-θ network). For each single-layer network, a data-driven thresholding method was employed by maximizing the global cost efficiency vs. the cost of the surviving functional connections (Bassett et al., 2009).

**Figure 1:**
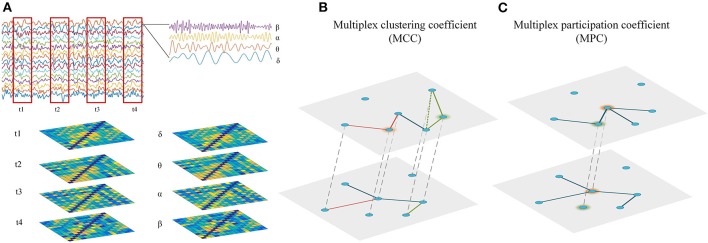
Reconstruction and analysis of cross-frequency or time-varying networks. **(A)** Reconstruction of multiplex networks. The frequency-based multiplex network (cross-frequency network) were constructed by integrating the four frequency-specific networks, while the time-dependent multiplex network (time-varying network) was constructed by integrating the time-specific networks within frequency bands. Each layer shared the same set of nodes and only the interactions between same nodes across layers were allowed. **(B)** Multiplex metrics of functional segregation. Multiplex clustering coefficient (MCC) was applied to assess local information processing in the multiplex networks. The green and orange nodes indicate MCC = 0 and MCC = 1, respectively. **(C)** Multiplex metrics of functional integration. Multiplex participation coefficient (MPC) was employed to assess global information processing in the multiplex networks. The green and orange nodes indicate MPC = 0 and MPC = 1, respectively.

As the static network analysis reflects the average behavior of the complete EEG recordings, we miss moment-to moment fluctuations in functional connectivity that might be informative about the brain states. Therefore, the connectivity matrices of time-dependent multiplex network were computed using a sliding time window technique. As illustrated in Figure 1A, the time series of EEG are segmented into non-overlapping time windows of width 4 s, functional connectivity is assessed in each window and thus we can generate a multilayer network, where each layer denotes a certain time point. In this study, the time-dependent networks were reconstructed in each frequency band.

### Multiplex Network Analysis

#### Multiplex Metrics of Functional Segregation

The functional segregation of a network reflects local information processing and is usually characterized by a measure named clustering coefficient (Battiston et al., 2014). In a single-layer network, the clustering coefficient of a node is the proportion of connections among a node's neighbors and describe how close a node's neighbors tend to cluster together. For the weighted network, it can be defined as:

(4) $$\begin{array}{l} C_{i}=\frac{1}{k_{i}\left(k_{i}-1\right)}{\underset{j,k}{\sum }\left(w_{ij}w_{jk}w_{ki}\right)}^{1/3} \end{array}$$

where *k*_*i*_ is the degree of node *i* in the corresponding binary network and *w*_*ij*_ is the link weight between nodes *i* and *j*. In fact, Equation (6) contains information about the fraction of triads centered in *i* that close into triangles and the weight of edges in the triangles. Averaging the measure over all nodes, one can obtain the network clustering coefficient.

In the multiplex networks, many nodes may display different clustering coefficient values across the layers. However, calculating the measure for each layer tells us little about the interplay between layers. Therefore, we need a multiplex measure to explore the formation of triangles by edges in different layers. Similar to the definition of clustering coefficients for node-aligned multiplex binary networks in the study performed by Battiston et al. (2014), we can express the multiplex clustering coefficient (MCC) for each node as:

(5) $$\begin{array}{l} C_{i,1}=\frac{\sum_{\alpha }\sum_{\kappa \neq \alpha }\sum_{i\neq m,j}{\left(w_{ij}^{\alpha }w_{jm}^{\kappa }w_{mi}^{\alpha }\right)}^{1/3}}{\left(M-1\right)\sum_{\alpha }k_{i}^{\alpha }\left(k_{i}^{\alpha }-1\right)} \end{array}$$

where *M* is the number of layers and $w_{ij}^{\alpha }$ is the link weight between node *i* and *j* in layer α. It quantifies the fraction of triangles where edge *j* − *m* belongs to layer α when the other two edges *i* − *j* and *i* − *m* belong to a second layer in terms of link weights. Similarly, a second weighted clustering coefficient for multiplex networks could be defined as a fraction of triangles where the three edges belong to different layers when the edges *i* − *j* and *i* − *m* belong to layers α and κ, respectively:

(6) $$\begin{array}{l} C_{i,\text{2}}=\frac{\sum_{\alpha }\sum_{\kappa \neq \alpha }\sum_{\mu \neq \alpha ,\kappa }\sum_{i\neq j,m}{\left(w_{ij}^{\alpha }w_{jm}^{\mu }w_{mi}^{\kappa }\right)}^{1/3}}{\left(M-\text{2}\right)\sum_{\alpha }\sum_{\kappa \neq \alpha }\sum_{i\neq j,m}{\left(w_{ij}^{\alpha }w_{mi}^{\kappa }\right)}^{1/3}} \end{array}$$

Notably, *C*_*i*,1_ is a suitable definition in the cases *M* ≥ 2, while the second definition requires that the network is composed of at least three layers. Hence, only *C*_*i*,1_ is considered in this study as a direct measure of network segregation, which may reflect local information processing in the multiple brain networks. If a triangle can be formed with any edges centered in node *i* and the edges belong to two layers, *C*_*i*,1_ is equal to 1. Conversely, if no triangle can be formed with edges centered in node *i* or the triangle can be formed only by the edges in one layer, *C*_*i*,1_ is equal to 0 (Figure 1B). Correspondingly, the multiplex clustering coefficient of the network can be formed as:

(7) $$\begin{array}{l} C=\frac{1}{N}\overset{N}{\underset{i\;=\;1}{\sum }}C_{i,1} \end{array}$$

Similar to the clustering coefficient in the single-layer network that describes the tendency to form locally dense clusters or modules, MCC can be applied to characterize the tendency of network nodes to form locally connected triangles across different layers. Therefore, higher MCC values may indicate increased efficiency of information flow in corresponding clusters (local brain regions) across frequency bands or time.

#### Multiplex Metrics of Functional Integration

In a multiplex network, though two nodes have the same value of overlapping degree, they may play different roles considering degree distribution across layers. To characterize the functional integration of connections in different layers, we employed a measure named multiplex participation coefficient, which quantifies the participation of a node to different layers. It can be expressed as:

(8) $$\begin{array}{l} p_{i}=\frac{M}{M-1}\left[1-{\overset{M}{\underset{L\;=\;1}{\sum }}\left(\frac{k_{i}^{L}}{o_{i}}\right)}^{2}\right]\text{=}\frac{M}{M-1}\left[\text{1-}{\overset{M}{\underset{L\;=\;1}{\sum }}\left(NLP_{i}^{L}\right)}^{2}\right] \end{array}$$

where *o*_*i*_ is the overlapping degree of node *i* ($o_{i}=k_{i}^{1}+k_{i}^{2}+\ldots +k_{i}^{M}$) and NLP is the node-degree layer proportion, which measures whether the links of node *i* are uniformly distributed in different layers. If the node has the same degree in all layers, *P*_*i*_ is equal to 1. On the contrary, if the links of node *i* are concentrated in one layer, *P*_*i*_ is equal to 0 (Figure 1C). For the frequency-based multiplex networks, this measure describes the link distribution in different frequency bands. While for the time-dependent networks, it depicts the fluctuation of node degree over time. From a statistical perspective, nodes with high *P*_*i*_ are considered central hubs as they allow better information exchange across different frequency bands or time points. The multiplex participation coefficient (MPC) of the whole network is the mean value of *P*_*i*_ over all nodes:

(9) $$\begin{array}{l} P=\frac{1}{N}\overset{N}{\underset{i\;=\;1}{\sum }}P_{i} \end{array}$$

Different from the participation coefficient describing integration of different modules or communities, MPC denotes the heterogeneity of connectivity patterns (nodal degree distribution) in each layer. From a statistical view, a random walker reaching nodes with high MPC values will jump to any other layers with similar properties. Hence, higher MPC values may facilitate the global information processing across layers with increased efficiency.

### Statistical Analysis

We first analyzed the multiplex network features on global scales (network level) to detect statistical differences between AD patients and healthy controls. Then, we assessed possible group difference in single nodes (local scale). We applied Mann–Whitney *U*-test to assess statistical differences of network metrics between groups with a significance level of 0.05. Before this analysis, the multiplex graph features were averaged over the epochs for each subject. As the analysis was conducted in different bands or nodes, we computed an adjusted version of the false discovery rate (FDR) for multiple comparisons as a post correction. The statistical significance level was set at *p* < 0.05.

### Classification Analysis

To further assess the possible application of multiplex network metrics in detecting AD, we implemented classification analysis based on the multiplex graph features on local scales. Considering the fact that high-dimensional input may increase the computational cost and lead to overfitting in the classification, we applied feature extraction and selection techniques to improve the classification performance. Only the features with significant group difference were considered in the classification process. After that, the least absolute shrinkage and selection operator (LASSO) logistic regression algorithm was applied to select the most significant predictive features and remove redundant features. By combining LASSO and multifactor logistic regression, the regression coefficients of most features were set to zero and the features with non-zero coefficients were preserved. Therefore, LASSO is suitable for reduction and selection of high-dimension features.

We used a classification approach to evaluate the performance of local network features in discriminating AD patients from healthy controls. In this study, the non-linear support vector machine (SVM) with a radial basis function kernel was employed as a classifier. Before the training process, the dimension reduction was done by the feature selection technique. Then, the 10-fold cross validation was performed to obtain more robust classification rates. To assess the classification performance, we computed the classification accuracy, sensitivity, and specificity. Receiver operating characteristic (ROC) curve was used to test the diagnostic ability of the classifier with varying thresholds, and the corresponding area under the curve (AUC) was also calculated. Since the multiplex networks may consist of different number of layers, we test different combinations of features in the classification process.

## Results

### Integration and Segregation of Cross-Frequency Network

**Figure 2:**
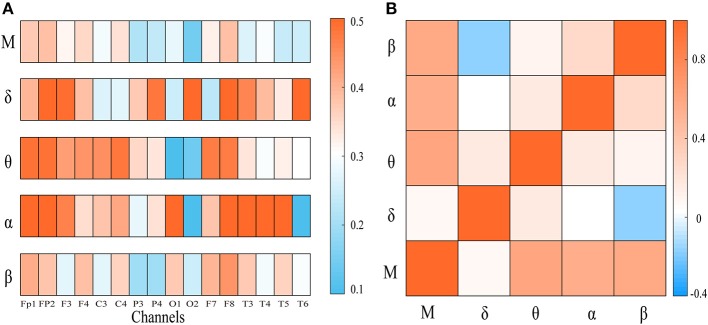
**(A)** The node clustering coefficient of the frequency-specific networks and cross-frequency networks for a representative patient. **(B)** The heat map represents the correlation of node clustering coefficient sequence between frequency-specific networks or between frequency-specific and cross-frequency networks. “M” denotes frequency-based multiplex network. Notice that the node clustering coefficient in multiplex network may be uncorrected or weakly correlated with that in frequency-specific networks.

To explore the integration of all frequency components, we first computed the weighted clustering coefficients of the four-layer multiplex networks at both global and local scales. In Figure 2A, the clustering coefficient of each node in frequency-specific networks was presented for an AD patient together with MCC of the four-layer networks. As shown, many nodes display quite different values of clustering coefficient across the frequency bands. We also computed the Pearson correlation coefficient of clustering coefficient between each single-layer network and between single-layer and multi-layer networks shown in Figure 2B. Notice that the sequences of clustering coefficient in different layers (band) are uncorrected or weakly anti-corrected, while MCC may show no correlation or weak correlation with clustering coefficient in frequency-specific networks. This result confirms that MCC may provide important information different from those obtained within frequency bands.

**Figure 3:**
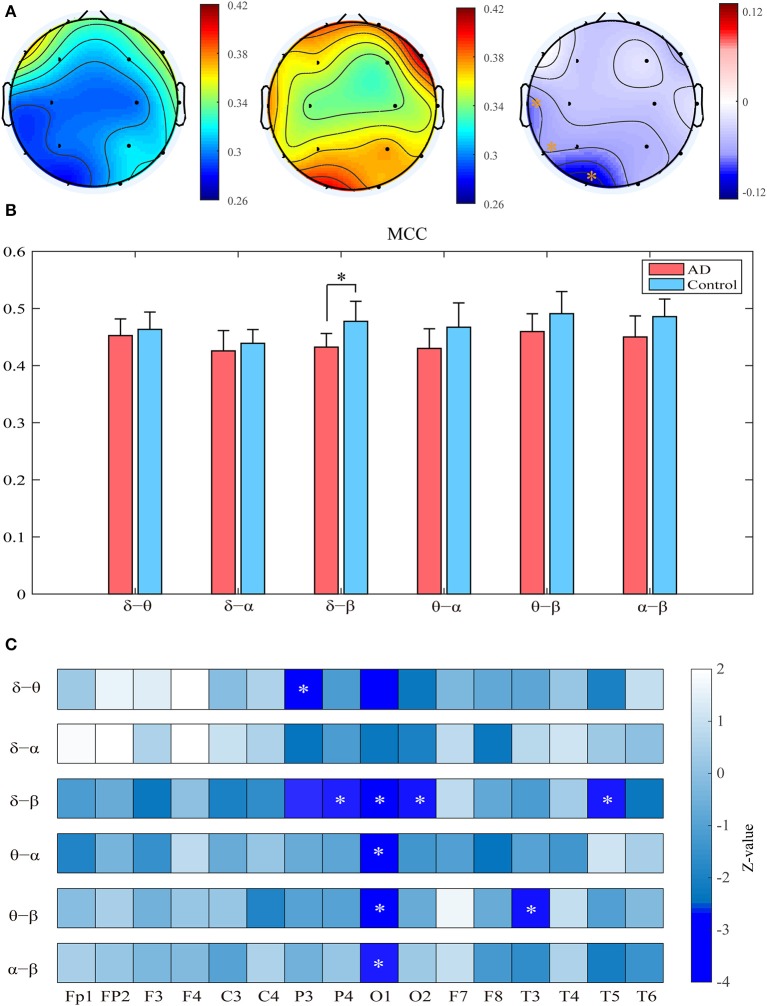
Functional segregation of the cross-frequency networks. **(A)** Topological distribution of MCC (averaged over the subjects) for both groups and their difference in the four-layer networks. **(B)** Averaged MCC in the two-layer networks at global (network) level for both groups, with vertical bars indicating group-averaged values while error bars denote standard errors. **(C)** Group difference of MCC in the two-layer networks at the node level denoted by *z* values, which are computed by Mann–Whitney *U*-test. The asterisks indicate significant group difference (*p* < 0.05, FDR corrected).

Compared with the controls, the patients show significantly decreased MCC in the left occipital and temporal regions compared to the controls (*p*_*FDR*_ < 0.05 with Mann–Whitney *U*-test). Most of these regions had relatively high clustering coefficient in the controls. We further computed the clustering coefficients of two-layer (i.e., δ-θ) networks to investigate the local information processing between two frequency bands (Figure 3). For the two-layer networks, the clustering coefficients in AD are significantly declined in the δ-β network in the central, parietal, and occipital areas (*p*_*FDR*_ < 0.05 with Mann–Whitney *U*-test). At the local scale, it is noteworthy that most of these networks show declined clustering coefficients in the occipital area, which may be a critical node responsible for the impaired local information processing across frequency bands in AD.

**Figure 4:**
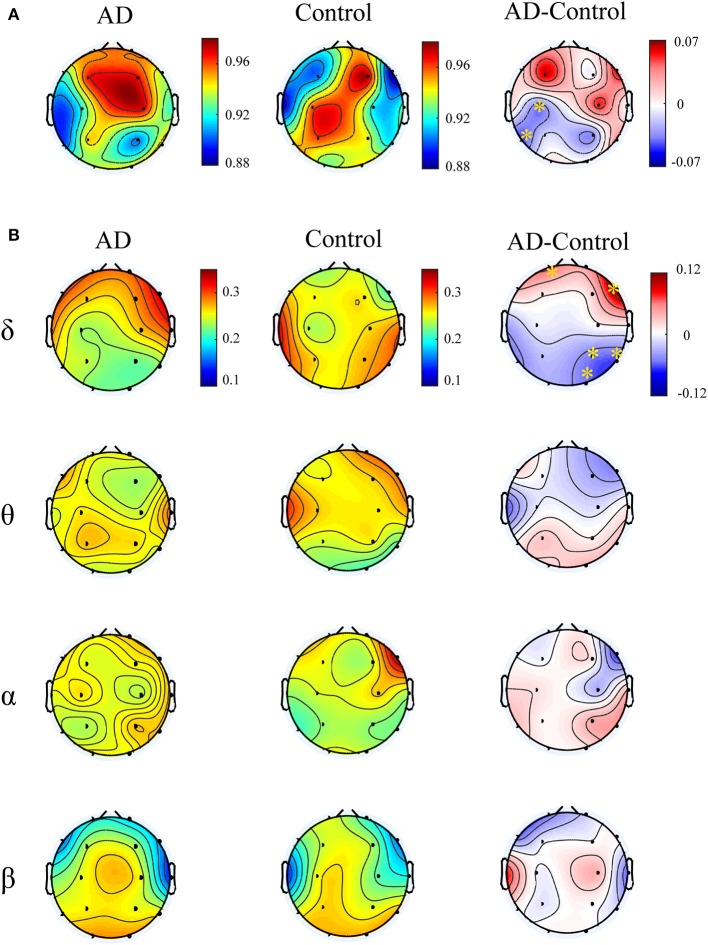
Functional integration of the cross-frequency networks. **(A)** Topological distribution of MPC (averaged over the subjects) for both groups and their difference in the four-layer networks and **(B)** the corresponding node-degree layer proportion (NLP) in each frequency band. The asterisks indicate significant group difference (*p* < 0.05, FDR corrected).

Figure 4A illustrates multiplex participation coefficient in different brain areas averaged over AD patients and healthy controls separately. Both groups exhibited high multiplex participation coefficients (MPC > 0.88) in most brain regions, suggesting a general propensity of human brain to promote information exchange across different rhythms. Interestingly, such tendency may vary among different regions and between groups. In comparison with the controls, AD patients show reduced MPC in the left posterior areas but increased MPC in the right frontal areas (*p*_*FDR*_ < 0.05 with Mann–Whitney *U*-test), where relatively high MPC values can be found in the patients. This implies that the brain regions may play different roles in the information passing across frequency bands. In AD, the increase of MPC in the frontal areas may compensate for the decreased MPC in the posterior areas to maintain essential information communication in the multiplex networks.

We also compared the node degree proportion (NLP) in all bands, which describes the within-frequency information exchange in the brain (Figure 4B). Notably, both groups show heterogeneous distribution of node degree among different brain regions and frequency bands, leading to the distinct spatial distribution of MPC between groups. For instance, in the delta band, the patients exhibit higher NLP in the frontal but lower NLP in the posterior areas (similar to the difference of MPC), suggesting that global information processing within bands also differs among different brain regions for both groups. Moreover, significant group difference is found in partial brain regions with decreased or increased NLP in delta and beta bands (*p*_*FDR*_ < 0.05 with Mann–Whitney *U* test). However, the regions with remarkable group difference of MPC may show no difference of NLP in any band. This confirms that the global information exchange across bands are determined by the integration of frequency-based multiplex networks rather than the network in a certain band.

### Integration and Segregation of Time-Varying Network

**Figure 5:**
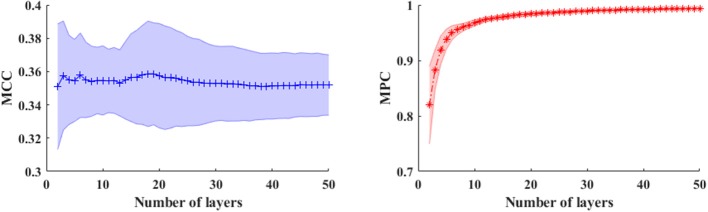
The averaged MCC (left panel) and MPC (right panel) in time-varying networks (in broadband) across the subjects with different number of layers (window length is 4 s). The shaded areas denote the standard deviation of metrics.

In a multiplex framework, we also investigate the temporal dynamics of brain networks via the multiplex network metrics within different frequency bands. For instance, the multiplex clustering coefficients can be considered as a dynamic character of tendency of nodes to form triangles between two time points. In the multilayer network framework, the performance of these measures are mainly influenced by number of layers (NL) especially in the time-varying networks. To illustrate this effect, we calculated the measures in time-varying networks with different layers shown in Figure 5. Results showed that number of layers (time length) has little influence on MCC, but the MPC values are dramatically elevated first (NL < 6) and then slightly increased to 1. This indicates that the MPC measure is suitable for the analysis within short time ranges (small number of layers) but unable to detect the changes in long ranges. Therefore, the number of layers is set to 5 for the analysis of time-varying networks.

**Figure 6:**
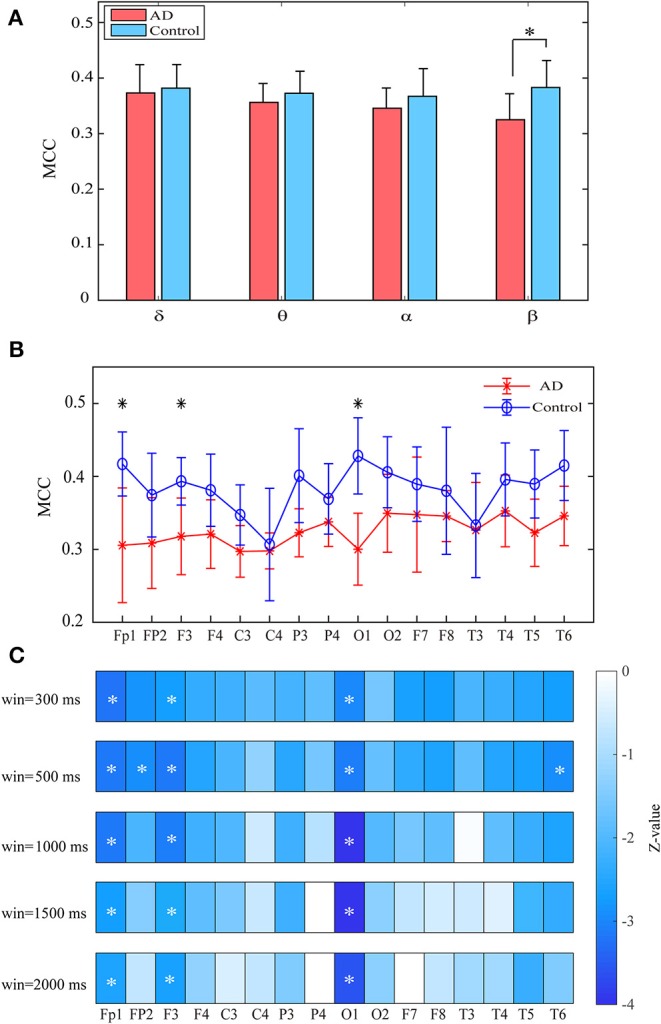
Functional segregation of the time-varying networks within frequency bands. **(A)** Averaged MPC over the subjects in different bands for both groups at global level. **(B)** Averaged MPC over the subjects in beta band for both groups at node level. **(C)** Group difference of MPC in beta network at node level (denoted by *z* values) with different window length. The asterisks indicate significant group difference (*p* < 0.05, FDR corrected).

Both groups show uniformly distributed MCC values among brain regions. In comparison with the control subjects, AD patients exhibit remarkably decreased MCC in the beta band, particularly in the frontal and occipital areas (*p*_*FDR*_ < 0.05 with Mann–Whitney *U*-test), indicating that the local information processing is also disrupted from a dynamic perspective (Figures 6A,B). We also computed the MCC values of the beta network with different window sizes, which may influence the results of statistical analysis, as shown in Figure 6C. Results show that both groups show similar spatial distribution of MCC when window size changes from 0.3 to 2 s and the statistical analysis results also reached a consensus with different window lengths.

**Figure 7:**
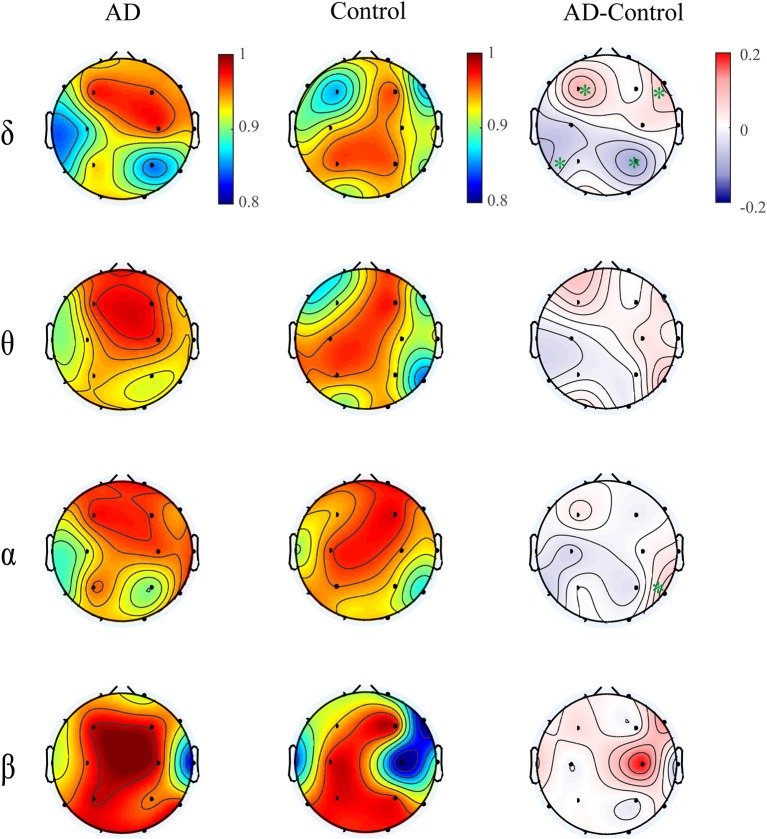
Topological distribution of MPC averaged over the subjects for both groups and their difference in the time-varying networks within frequency bands. The asterisks indicate significant group difference (*p* < 0.05, FDR corrected).

Moreover, we computed the multiplex participation coefficient for the time-dependent networks (Figure 7). Since the MPC in frequency-integrated networks depicts the information exchange across frequency bands, the MPC in time-dependent networks can be regarded as an indicator of the global information processing along time. The patients show higher MPC values in the frontal-central area than those in other regions, indicating that the frontal-central area may play a major role in the global information communication within bands over time. While for the controls, the MPC values are distributed more irregularly among regions. Such distinct spatial distribution between groups leads to an increased trend of MPC in the right frontal area and decreased trend in the left posterior area for AD in most frequency bands, though the group difference may not be significant. These results suggest that the node degree distribution of brain networks fluctuates with time and such fluctuations differ among brain regions and between groups. On the other hand, the alteration of MPC in the time-dependent networks imply that AD brain may operate at a less optimal point in terms of the global information processing along time.

### Classification Analysis Results

**Figure 8:**
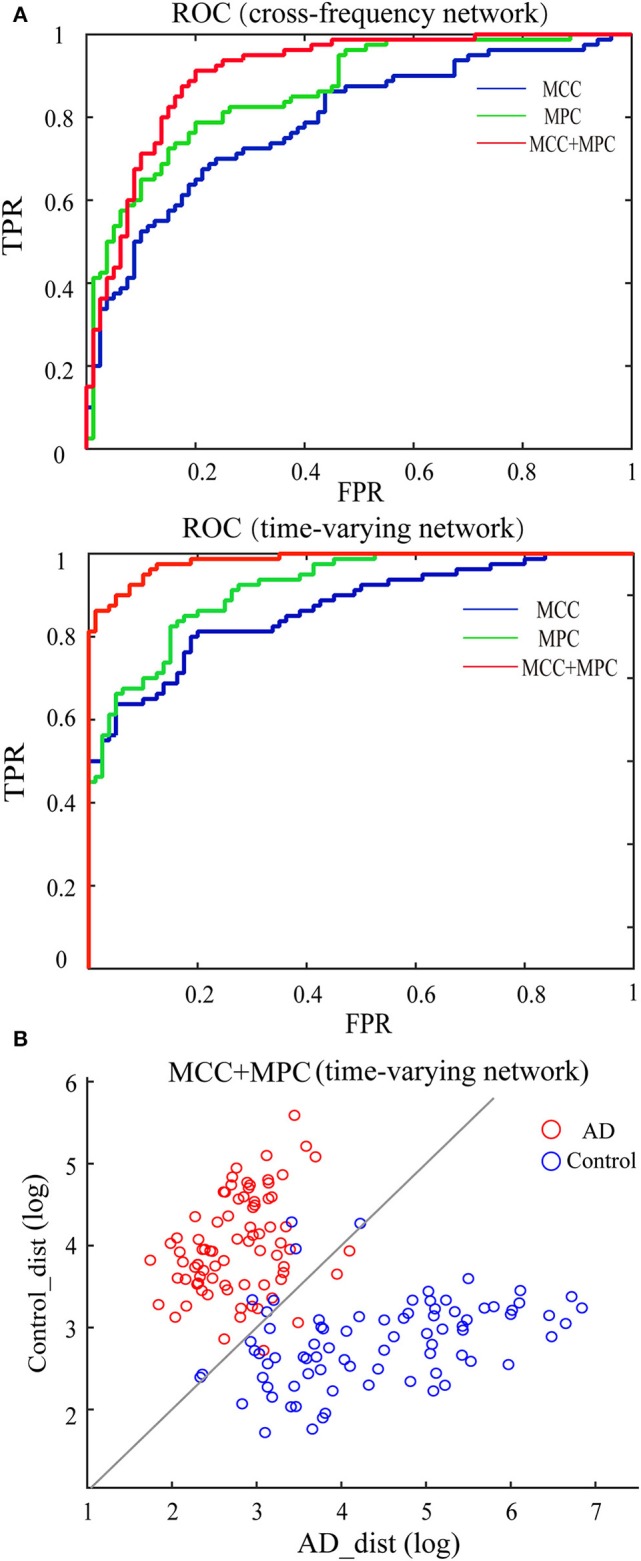
Classification performance of the multiplex network features at node level. **(A)** ROC analysis with the features of cross-frequency networks (top) and time-varying networks (bottom). **(B)** Scatter plot shows the Mahalanobis distance of each sample from AD or control class with the combination of MCC and MPC in the time-varying networks (gray line indicates equal distance). The best performance was achieved by the combination of MCC and MPC in the time-varying networks.

We compared the classification performance obtained by different combinations of features, as shown in Figure 8A and Table 1. Note that the feature selection procedure was conducted separately for each combination before the classification experiments. In the cross-frequency networks, an average accuracy of 70.63% was achieved by the MCC (AUC = 0.78), while for the MPC, the classification performance was much better with an accuracy of 77.5% and an AUC of 0.86. By combining the two features, the average accuracy and AUC improved to 82.5% and 0.90, respectively. Compared to the classification results in terms of inter-frequency dynamics, the classification performance was remarkably enhanced when employing the multiplex network features related to temporal dynamics. The combination of MCC and MPC in the time-varying networks achieved the best performance (accuracy = 92.5%, AUC = 0.98), which was further confirmed by the scatterplot of the Mahalanobis distance of each sample (epoch) from AD or control class depicted in Figure 8B.

**Table 1:** Classification performance of multiplex networks features via the SVM classifier.

	**Features**	**Accuracy (%)**	**Sensitivity (%)**	**Specificity (%)**	**AUC**
Cross-frequency	MCC	70.63	72.50	68.75	0.7884
	MPC	77.50	82.50	72.50	0.8638
	MCC+MPC	82.50	78.75	86.25	0.9066
Time-varying	MCC	79.38	77.50	81.25	0.8669
	MPC	81.25	88.75	73.75	0.9173
	MCC+MPC	92.50	96.25	88.75	0.9836

We further compared the classification performance with other classifiers, including linear SVM, k-NN, naive Bayes, and Fisher discriminant analysis classifier, in which the combination of MCC and MPC in the time-varying networks was regarded as the input of each classifier. The classification results with all the classifiers are listed in Table 2. It is demonstrated that all the classifiers exhibit promising results with over 90% accuracy. In particular, the SVM and discriminant analysis classifier show similar results with an averaged accuracy of 92.5%.

**Table 2:** Classification performance of the time-varying network features via different classifiers.

**Classifier**	**Accuracy (%)**	**Sensitivity (%)**	**Specificity (%)**
SVM_RBF	92.50	96.50	88.75
SVM_linear	91.87	95.00	88.75
NB	90.63	91.25	90.00
FDA	92.5	93.75	91.25
KNN	90.63	92.50	88.73

## Discussion

In this study, we investigated the inter-frequency and temporal dynamics of functional networks in patients with AD. By integrating the frequency-specific networks or time-varying networks with different connectivity patterns, we explored the alteration of the local and global information processing across frequency bands or time in AD. Compared to the controls, the AD brain was characterized by impaired local information processing in both frequency-based and time-dependent multiplex networks, while for global information processing, the alterations may differ among different brain regions in which an opposite trend can be observed. The classification analysis further confirmed that the multiplex network metrics could be used to distinguish AD patients between controls, and the best classification performance was achieved by combining multiplex clustering coefficients and participation coefficients of time-varying networks.

### Inter-frequency Dynamics of Brain Networks

Network integration and segregation has conventionally been analyzed in different frequency bands, as the wide range of frequencies are considered to be associated with different cognitive processes. Previous studies related to AD have shown that functional connectivity patterns in EEG networks are frequency-specific (de Haan et al., 2012; Hillebrand et al., 2016; Yu et al., 2016). Here, we integrate the four frequency-specific networks in a multiplex network framework. Multiplex clustering coefficients and participation coefficients were employed to investigate the network integration and segregation across frequency bands. The interaction of different rhythms has been reported to play a critical role in the cognitive process (i.e., working memory) and show difference in different brain states (Tewarie et al., 2016; Shi et al., 2019). Therefore, network study in view of inter-frequency dynamics can offer new insight into the local and global information communication in human brain.

Our results showed decreased segregation in the AD group at the global network level, particularly in the multiplex networks integrating beta and other bands; thus, we can speculate that the local information transformation between frequency bands is disrupted in AD brain and the beta rhythm occupies an important position in the modification of network segregation by AD. We also found that left occipital area (channel O1) in AD shows a remarkable decrease of MCC in most cross-frequency networks (i.e., δ-θ network), suggesting that this area may play an essential role in the local information exchange between different frequency bands and such exchange may be selectively impaired in AD patients.

As a metric quantifying the integration in a multiplex network, MPC describes the global information exchange between different frequency-specific networks. It can be applied to evaluate the regional centrality of a cross-frequency network, as the nodes with high MPC values allow a random walker jump with similar probability to other layers and thus facilitate the information transmission across frequency bands. We found that AD brain may show decreased integration in the posterior area resulting from altered spatial distribution of MPC. This result is in line with a recent MEG study reporting the disruption of specific occipital hub regions in AD. During the progression of disease, the patients may exhibit decreased hub centrality or number of hubs (Yu et al., 2017). Alternatively, owing to the loss of original hubs, some non-hub areas may become relatively more important to maintain the information flow within or across frequency bands and even become new hubs. This modification is also confirmed by the increased MPC in the frontal area of AD brain.

A recent EEG network study has also investigated inter-frequency dynamics in AD using multi-layer network metrics (Guillon et al., 2017). They focused on the global information processing across all frequency bands, while in our study, we explored the information exchange between any two bands that may also show abnormalities in AD brain. Moreover, we combined the segregation and integration properties in the cross-frequency networks to get a more complete picture of the inter-frequency dynamics for AD. Within the multiplex network framework, we further investigated the information processing across time (discussed in the next section) using the multiplex graph metrics.

### Temporal Dynamics of Functional Networks Within Frequency Bands

The static graph analysis has proved to be effective in characterizing the disturbed functional connectivity in AD. Many studies investigating the clustering coefficient has showed that the AD brain is characterized by a decrease of clustering in different frequency bands, while other studies may show an opposite trend (Tijms et al., 2013). This inconsistency can also be found for the normalized clustering coefficient (relative to the random networks). As a metric reflecting global information processing, the participation coefficient is also widely used to measure the diversity of inter-modular connections (Kabbara et al., 2018). A reduction of gamma inter-modular connectivity has also been found in patients with AD (Guillon et al., 2017).

In the present study, we extended the framework of brain network analysis by the investigation of information communication in functional networks from a dynamic view. Multiplex clustering coefficient was employed as a dynamic graph metric to study the local information processing over time. Unlike the clustering coefficient of a static graph, the multiplex clustering coefficient describes how likely the nodes tend to cluster between any two time points. In fact, for a complex system, the local information communication between elementary entities may take a relatively long time rather than being completed within a short time window. Therefore, MCC can be applied to describe the properties of local information processing across time and provide additional information different from those obtained by looking at the clustering in a static network. In this study, both groups show uniform distribution of MCC among brain regions, implying that these regions may play different roles in the local specialized processing of information over time. Moreover, for the patients, significant decrease of MCC was found in the delta and beta bands, particularly in the left occipital area. This suggests that the local communication efficiency of the dynamic network is also selectively disrupted in AD.

Similar to the MPC of cross-frequency networks that depicts the heterogeneity of global information flow across frequency bands, MPC of the dynamic network reflects the temporal heterogeneity of network information exchange over time. The node areas with high MPC values are more likely to allow information flow across time. In our study, results exhibited that the efficiency of global information communication differs among brain regions, as both the patients and controls show spatial heterogeneity of MPC in all frequency bands. No significant group difference was found in the MPC at the global level when averaging MPC over all nodes, which can be partly attributed to the fact that the AD network may show an increased trend similar to that in cross-frequency networks. Therefore, the AD brain cannot be simply characterized by declined or enhanced information processing in the dynamic networks. Instead, the integration of information at the node level may show more significant difference among brain regions than that between groups. Such spatial difference may also relate to progression of the disease and needs further research.

### Limitation

One limitation of the present study is the small sample size of subjects. Our intent was to characterize the abnormalities of information exchange in the multiplex brain networks for AD patients. Nevertheless, we also recognized the fact that AD may have different stages and show heterogeneous characteristics among the patients. Moreover, tracking the change of brain activities from mild cognitive impairment (MCI) to AD is also an interesting topic in AD research and needs future investigation. These investigations should be performed on larger cohorts of patients with different cognitive levels and using other experimental paradigms.

Another limitation is that the influence of EEG reference on our results was not discussed. Previous studies have shown that the choice of EEG reference can affect functional connectivity estimation and the reference electrode standardization technique is proved to be a better choice (Yao, 2001; Lei and Liao, 2017). Though the reference choice may not have much effect on the group analysis results (e.g., classification performance) as it has on the connectivity estimation for single subject, future EEG studies should take the choice of EEG reference into consideration to achieve a more stable and robust performance.

## Conclusion

In this study, we provided an effective framework to study the functional segregation and integration of brain networks considering inter-frequency and temporal dynamics. In this framework, the patients show decreased segregation particularly in the occipital area, while the alteration of integration differs among brain regions in both cross-frequency and dynamic networks. These obtained results gave new insights into the abnormalities of information exchange in brain networks and may benefit our understanding of pathology for AD. On the other hand, reconstructing and analyzing the functional networks in a frequency-integrated or time-varying manner may enrich the methodologies about the deconstruction of brain activity underlying EEG. Future studies will involve the subjects with mild cognitive impairment and be extended to other neurodegenerative diseases.

## Data Availability Statement

The datasets generated for this study are available on request to the corresponding author.

## Ethics Statement

The studies involving human participants were reviewed and approved by the Ethics committee of Tangshan Gongren hospital. The patients/participants provided their written informed consent to participate in this study.

## Author Contributions

LC: manuscript drafting, data analysis, and interpretation. XW: design of study. JL: data collection. LZ: data analysis. JW and BD: manuscript revision. HY: design of study and manuscript revision. RW: data collection.

### Conflict of Interest

The authors declare that the research was conducted in the absence of any commercial or financial relationships that could be construed as a potential conflict of interest.
